# An Interpretive Phenomenological Analysis of Teachers’ Lived Experiences of Working with Traumatised Children in the Classroom

**DOI:** 10.1007/s40653-024-00614-9

**Published:** 2024-02-13

**Authors:** Antoinette Frearson, Mandy Duncan

**Affiliations:** School of Education, St John’s Campus, University of Worcester, Henwick Grove, WR2 6AJ Worcester, UK

**Keywords:** Childhood Trauma, Adverse Childhood Experiences (ACEs), Covid-19 Pandemic, Teacher Mental Health, Interpretive Phenomenological Analysis (IPA)

## Abstract

This study illuminates teachers’ lived experiences of working with traumatised children in school environments. Children who experience trauma display a range of behaviours in the classroom which impact on attainment and outcomes. Dealing with childhood trauma in the classroom is challenging and brings risks to teachers’ mental health including secondary traumatic stress and burnout. Interpretative phenomenological analysis (IPA) was employed to understand the lived experiences of teachers working with traumatised children in the classroom. Findings from in-depth semi-structured interviews with six teachers indicate that teachers increasingly support traumatised children in the classroom but there is a need for targeted trauma-informed training and effective support from senior management to support teachers’ mental health and wellbeing.

## Introduction

The purpose of this study is to illuminate teachers’ lived experiences of working with traumatised children in the classroom. It draws on a small sample of teachers who have varied experiences of working with children who have undergone a range of traumatic experiences including those related to conflict, abuse and the Covid-19 pandemic. It contributes to the scholarship on a topic which has assumed great significance given the recent mass displacement of children as a result of the conflicts in Ukraine and Gaza which have left millions of children at risk of long-term physical and mental health problems (Abudayya et al., 2023; Elvevåg & DeLisi, 2022). It is also particularly relevant in the wake of the Covid-19 pandemic which has seen high rates of negative mental health outcomes in children and young people (Sprang et al., 2023) and left teachers with the need for resources to help them cope with the increased stress and demands that supporting these children brings (McCarthy et al., 2022).

One in thirteen young people in the UK experience trauma before the age of eighteen, and as a consequence of this trauma, are twice as likely as their peers to suffer from a range of mental health disorders (Lewis et al., 2019). Childhood trauma is also associated with anti-social behaviours, lowered life satisfaction, chronic disease and higher costs of care across the life-course (Florence et al., 2013; Smith & Smith, 2010; Ulukol et al., 2016; Yang & Hedeker, 2020). Within the classroom, traumatised children might display a range of behaviours that can mask their fear, sadness or loss such as aggression, hyperactivity and problems with concentration, memory and self-regulation (Gabowitz et al., 2008; Kim et al., 2013). Negative outcomes can be minimised when teachers are able to identify trauma-related behaviours early and put support in place to allow children opportunities for recovery (Florence et al., 2013; Lowry et al., 2022; Smith & Smith, 2010; Ulukol et al., 2013). However, in the UK there is a lack of training and support for teachers to develop pedagogies that meet the needs of traumatised children, and this can erode their confidence, motivation and perseverance in undertaking this work (Pines, 2002). Sullivan et al. (2014) suggest that teachers who feel that they lack competency in supporting traumatised children successfully are more likely to experience burnout and leave the profession.

The challenge of supporting traumatised children has become even more prominent as a result of the Covid-19 pandemic and associated lockdowns, which increased the vulnerability of many children to abuse both online and within the home. Abuelezam (2020) goes on to explain how many other children experienced mental health problems following Covid-19 as a result of isolation, loneliness or the loss of a loved one. The research that we present here was carried out during the pandemic and adds to the existing literature by capturing teacher voice through the use of interpretative phenomenological analysis (IPA). It explores a small sample of primary and secondary teachers’ experiences of classroom trauma including how they identify and manage trauma in their classrooms, their experiences of trauma training and support, and their suggestions for how current practices could be improved.

### Childhood Trauma

The National Child Traumatic Stress Network (NCTSN) (2022a) describe childhood trauma as the emotional response to experiencing a frightening, dangerous or distressing event that poses a threat to a child’s life, bodily integrity or psychological wellbeing. It can also occur after witnessing an event that threatens the physical safety or security of a loved one. Copeland et al. (2007) estimate that around two thirds of children will experience at least one traumatic event before they reach adulthood. These traumatic events can be emotionally painful and may overwhelm a child’s ability to cope with everyday life; some children will develop stress reactions that persist and affect their daily lives long after the initial event or events have passed (International Society for Traumatic Stress Studies (ISTSS), 2022; NCTSN, 2022a).

The Adverse Childhood Experience (ACE) study found that over half of its 13,494 participants had experienced one ACE and a significant number had experienced two or more (Felitti et al., 1998). The study identified various types of ACE including psychological, physical or sexual abuse; domestic violence; living with a household member who is a substance abuser, mentally ill or suicidal; or having an incarcerated relative. A graded association has been found between the number of exposures to childhood trauma and increased risk-taking behaviours in adults such as: smoking, alcoholism, drug abuse, promiscuity, depression and severe obesity (Binder, 2009; Felitti et al., 1998). An increased risk of various medical conditions has also been associated with ACEs including ischemic heart disease, cancer, chronic lung disease, skeletal fractures, and liver disease (Center for Disease Control and Prevention (CDC), 2022; Felitti et al., 1998). The ACE study has done much to raise awareness about the potential consequences of childhood experiences on long-term outcomes, however, there have been various criticisms of the study including the design of the questionnaire which conflates experiences and can encourage a reductionist view of a very complex issue (McLennan et al., 2019). It is also important to note that the findings should not be taken to imply causation and extrapolations cannot be made as individuals who suffer traumatic experiences as a child do not necessarily experience associated health or social problems. A range of confounding factors might also impact on life outcomes such as socio-economic status, poverty and discrimination and these have later been added to the framework for understanding ACEs within the broader community context (Diab & Schulz, 2021; Ellis & Dietz, 2017).

### Pandemic Related Trauma

The Covid-19 pandemic has been described as an unprecedented and unparalleled traumatic stressor which has had wide-ranging effects on both adults and children, particularly those with pre-existing mental health conditions (Bridgland et al., 2021; Campion et al., 2020; CDC, 2022; Janiri et al., 2021). It brought a range of stressful experiences to families across the world including billions of job losses, increased financial insecurities, care-giving challenges and isolation-related anxieties putting many children at an increased risk of experiencing trauma (Collin-Vézina et al., 2020; Guessoum et al., 2020; Prime et al., 2020).

In March 2020, schools closed in 188 countries around the world affecting more than 1.7 billion students with many children and young people having to be educated at home through online learning and by their families (Gallegos et al., 2022; United Nations Educational, Scientific and Cultural Organization (UNESCO), 2020). Recent studies have highlighted the significant impact of the lockdowns on children’s health and wellbeing as a result of experiencing social isolation, confinement and school closures in the UK (Fegert et al., 2020; Lawson et al., 2020; Rosenthal & Thompson, 2020) and across the world (Janiri et al., 2021; Kontoangelos et al., 2020; Pappa et al., 2020).

The financial and social vulnerability of many households during the pandemic increased the risk of various interpersonal and intrapersonal traumas such as physical and emotional neglect; family issues such as substance abuse, alcoholism and the worsening mental health of a parent or carer; physical, sexual and psychological violence; inter-parental violence and an increased risk of domestic abuse (Cénat & Dalexis, 2020; Substance Abuse and Mental Health Services Administration (SAMHSA), 2021). The continued impact on children’s mental health post-pandemic has affected school attendance, behaviour, and learning (Department for Education (DfE), 2022a).

### Conflict Related Trauma

Over the last fifteen years, instances of war and armed conflicts in the world have been steadily increasing (Institute for Economics and Peace, 2022). Save the Children (2021) report that one in six children in the world live within conflict zones in countries such as South-Sudan, Yemen, Ethiopia, Syria, Afghanistan, Ukraine and Gaza. Thousands of children die each year due to war-related violence such as bombing and millions more die from war’s indirect effects such as the death of carers, loss of homes, water system failure, and lack of food, sanitation and health services (Jordans et al., 2016; Veenema, 2001). Others are abducted from schools, homes or refugee camps and exploited for trafficking, sexual slavery and forced labour (Prasad & Prasad, 2009). These conditions enforce mass displacements and migrations subjecting children to further stress and re-traumatisation as they attempt to move to a safe place, sometimes alone and separated from their families (Khan & Khan, 2017; Miles et al., 2019; World Health Organisation (WHO), 2022). When refugees settle they are at risk of acculturation trauma and may experience discrimination; low socioeconomic status; a lack of social support; language and communication barriers; difficulties finding employment; fear of being forced to return or be moved on to an unsafe environment; loneliness; and fear for relatives left behind (DeJong et al., 2017; Pieloch et al., 2016; Safdar et al., 2021; von Haumeder et al., 2019). These experiences leave refugee children at increased risk of psychological trauma and mental health problems including post-traumatic stress disorder (PTSD) (Veenema & Schroeder-Bruce, 2003).

### Supporting Traumatised Children in School

A range of studies have documented the impact of childhood trauma within the classroom and the effect on educational outcomes. These have been shown to include poor school attendance; poor progress and attainment; effects on memory; learning and development; emotional and behavioural control; and anti-social behaviours (Bellis et al., 2017; Bethell et al., 2014; Duke et al., 2010; Perfect et al., 2016; Stempel et al., 2017). A systematic review of 83 studies found that students with cumulative or severe exposure to traumatic events are at higher risk of severely impaired cognitive functioning, academic difficulties, and socio-emotional behaviour problems (Perfect et al., 2016). CDC (2023) argue that the majority of children who experience childhood trauma can be supported to develop resilience which enables them to adapt to events that threaten their development and continue positively after one or more periods of adversity.

Providing children with educational, emotional and practical support, including neurological repair interventions such as mindfulness training, have been shown to be helpful in mitigating the impact of early-life stress (Bryck & Fisher, 2012). Chafouleas et al. (2016) suggest that schools are the ideal setting to assist the recovery of traumatised children through delivery of instructive interventions within stable systems of support. However, questions have been raised regarding the extent to which this is the responsibility of teachers and whether teachers feel competent in undertaking this role (Alisic, 2012; Reinke et al., 2011). Lowry et al. (2022) found that in the UK only 40% of teachers feel equipped to support children with mental health needs and that over half were not confident in supporting traumatised or grieving children following the Covid-19 pandemic. This is particularly problematic given that external support services cannot keep up with demand and many children referred by schools for mental health services face long waits. In England NHS Digital (2018) reported that only 25.2% of referred 5–19-year-olds were able to access a mental health specialist in 2017. This places schools and teachers under immense strain to provide the mental health support that children need but without appropriate professional training.

### Trauma Support and Training for Teachers

The statutory guidance for schools and colleges by the DfE (2022a) states that teachers have a professional responsibility to safeguard children by being aware of how experiences of childhood trauma can impact on their behaviour, mental health and education. The guidance also states that a history of childhood trauma and adversity can leave a child vulnerable to the risk of further harm and educationally disadvantaged in overcoming barriers such as behaviour, mental health, school attendance and learning; therefore, it is important that children are offered effective and appropriate support (DfE, 2022a). This is clearly high stakes work which can place significant demands on teachers’ time, knowledge and professionalism (Walsh & Farrell, 2008). Nonetheless, teachers play a key role in identification, successful management and recovery support for traumatised children within the classroom (Pachter et al., 2017). Bernard and Walton (2011) argue that schools must become involved in early intervention work by supporting staff to recognise signs of trauma in children, implementing trauma-informed classroom practices and creating robust policies to support positive behaviour and resilience. Reinberg and Fefer (2018) also argue that school trauma services should be intervention-focused, data-based and involve the whole school community. However, schools can be reluctant to identify and manage trauma due to concerns about over-reporting and uncertainties among teachers in how to respond to disclosures (Alisic, 2012; Reinke et al., 2011). Basic knowledge of trauma symptoms, behaviours and strategies are not generally included in initial teacher training courses however, Sinclair, Taylor and Hodgkinson (2001) maintain that there is a need for this and also for continuing professional development courses which together would provide layered training and support for teachers. Lowry et al. (2022) go further and show that teachers are providing essential support services for children whose needs are not being met by health services and therefore schools should receive funding to support them to employ and train existing staff to perform these public health and primary care roles.

### Teachers’ Mental Health

Teachers face enduring and mounting challenges including limited time for planning, limited administrative support, low salaries and limited opportunities for collaborative teaching or professional advancement. They are required to balance academic and disciplinary expectations and may find themselves dealing with uninterested students, parental conflicts, high class sizes, and constant change (Johnson et al., 2005; Kyriacou, 2001; Organisation for Economic Co-operation and Development (OECD), 2014). On top of these daily challenges, teachers can find themselves deeply affected by children’s disclosures of daily hardships and adverse experiences of abuse, neglect, parental separation, danger and poverty. Teachers often have a great deal of compassion towards the children in their care but can also feel overwhelmed and powerless to address children’s suffering. This can lead to emotional detachment, compassion fatigue and secondary traumatic stress which results from listening to narratives of first-hand trauma (Hodgkins, 2019; NCTSN, 2022b; Thomas, 2013; Yang, 2021).

Symptoms of secondary traumatic stress include emotional numbness, depersonalisation (feeling at a distance), and a reduction in working memory; all factors which could potentially impact the quality of teaching (Bride et al., 2004; Bride, 2007). Teachers also report physical, emotional and behavioural changes, interpersonal isolation, cognitive dysfunction and diminished performance (Hydon et al., 2015). Other symptoms include recurring images of victims or intrusive thoughts about the traumatised child, feelings of blame and self-doubt, emotional sensitivity and restlessness (Denne et al., 2019; Van Bergeijk & Sarmiento, 2006). In some cases, these types of symptoms can lead to psychological detachment between the teacher and the child which can mediate the professional relationship and result in decreased reporting of suspected child abuse (Hupe & Stevenson, 2019).

Stress and exhaustion have been found to be causal factors for teachers leaving the profession (Grant et al., 2019). A recent examination of the relationships between early childhood educators, ACEs and job-related stressors showed that the risk of burnout was on the rise (Grist & Caudle, 2021). Hart and Nash (2020) and Klusmann et al. (2023) have reported higher levels of compassion fatigue among teachers during the Covid-19 pandemic resulting from an increased awareness of the traumas experienced by children and sustained efforts to re-engage children in learning after the lockdowns. The risks of teachers leaving the profession due to compassion fatigue, secondary traumatic stress and burnout are reduced when teachers have positive relationships with children and colleagues, support from their supervisor, a cohesive team, and an inspiring climate within which to work (Jansen in de Wal et al., 2020).

## Methodology

This study was underpinned by interpretative phenomenological analysis (IPA) which is a qualitative approach to understanding the lived experiences of individuals in their own terms rather than through existing theoretical frameworks. The aim, in choosing IPA as an approach, was to present an authentic representation of participants’ lived experiences (Thurston, 2014). IPA has been described as a means for collecting rich experiences of ‘being’ (Ravn & Christensen, 2014); in the case of this study, the experience of ‘being’ a teacher and interacting with children’s manifestations of trauma in the classroom. Teachers experiences of working with traumatised children are complex, diverse and emotionally laden, making IPA particularly suitable as it supports sense-making of a range of disparate accounts.

Critics of IPA hold that it lacks neutrality and objectivity and is overly subjective and emotional (Gardner et al., 2017). Arguments include that it leads to compromised, biased and flawed data which lacks rigour both analytically and methodologically (Creswell, 2003). However, all methods involve the researcher’s intentions, decisions and values, and these inevitably shape processes and decisions throughout any project (Gardner et al., 2017). The rationale for using IPA was to get as close to the experience as possible, not to remain distant from it, and this allowed us to make a detailed analysis of the perceptions of the participants and ensured that our interpretations were fully grounded in the data. We adopted a reflexive approach in order to reduce the risk of bias by examining the relationship between our positionality (as researchers and also educators ourselves) and the cognitive and affective perceptions of our participants. Following detailed and reflexive interpretation of the individual lived experiences of the participants, we employed inductive analogical reasoning to move to a more general understanding of the issue.

### Study Design

Following ethical approval from the University of Worcester, participants were each invited to take part in one semi-structured interview to gain as detailed accounts as possible of their individual experiences of interactions with children in the classroom who had undergone or were undergoing trauma. The interviews involved in-depth explorations of the perceptions, thoughts, feelings and motivations of teachers in relation to their experiences of managing childhood trauma within the classroom including their views on any support and training that they might have received in identifying and supporting traumatised children.

Semi-structured interviews allowed for open-ended questions to be planned but also offered flexibility for modification and further probing during the interviews (Given, 2008; Rabionet, 2011; Turner, 2010). Written consent was provided before the interviews took place and reassurance was given that if the interviews triggered any distressing memories or upset then they would be stopped and only recommenced if the participant felt able to do so. Signposting to mental health services for extra support if needed was also offered at this point. The data was collected via the Zoom platform at the time when the UK was emerging from the third lockdown of the Covid-19 pandemic and just a few weeks after schools had re-opened for all children. This context undoubtedly influenced the responses of participants and made for compelling findings which provided a real-time snapshot of how Covid was affecting children and their teachers. The use of online media to collect data through interviews had positive and negative outcomes. On one hand, it was the most time-efficient and cost-effective way of maximising sample variety (Hanna, 2012) and it ensured compliance with ongoing Covid restrictions. On the other hand, there was possibly some loss of rapport, subtle body language and facial expression cues as described by Lo Lacono et al. (2016). There were also some connectivity issues where the screen would freeze or go blank causing conversations to be a little stilted at times. This did not appear to affect the data collection unduly, although it is possible that the participants might have explored some areas further if the interviews had been face-to-face. All interviews were recorded on Zoom and then transcribed verbatim. The transcripts were returned to the participants to review to offer maximum assurance that they were a true account of the participants’ experiences and to allow them to make any edits or deletions before analysis (Tallman, 2019).

### Participants

The study drew on the accounts of six participants who were purposively selected for the meaningful insight they could provide into the issue due to their combined years of experience and the different contexts within which they had worked with traumatised children. The small sample size is consistent with other IPA studies and was necessary to illuminate the phenomenon in as much depth as possible (Frechette et al., 2020). Heterogenous sampling was employed to attain maximum variation within the sample in order to gain broad and deep insights (Patton, 2015). Recruited teachers had experiences in a range of educational contexts as outlined in Table 1.

**Table 1:** Participant demographics

Participant Code	Role/s and educational setting/s	Experience	Years in the profession	M/F
**S1**	Principal, teacher and school counsellor in primary, middle school and kindergarten settings working with mainstream, special educational needs (SEN) and refugee children.	1973–2001: Teacher in Republic of Ireland primary school Teacher and Principal in Namibian schools during the time of the South African Border War (Namibian War of Independence) Primary teacher in UK school. 2004 - present: UK School counsellor pre and during Covid.	24 years	F
**C2**	Teacher in mainly secondary mainstream and special school settings with some primary, adult education and English as an additional language (EAL) experience.	UK suburban and semi-rural settings pre-and during Covid.	20 years	M
**T3**	Teacher and Teaching Assistant (TA) in primary and secondary settings.	UK inner-city primary and secondary schools pre and during Covid.	25 years	F
**K4**	Teacher in primary setting.	UK semi-rural primary school in UK pre and during Covid.	4 years	F
**M5**	Teacher and Head of religious education in secondary setting.	An inner-city secondary school in the UK.	25 years	F
**M6**	Retired Headteacher in Secondary settings.	Inner-city secondary schools in Northern Ireland during the 1970s - a time of heightened civil disturbance.	34 years	M

### Approach to Data Analysis

The process of interpreting the data involved engaging the ‘double hermeneutic’ (Giddens, 1987, pp 20–21) which is based on the notion there are two frames of meaning. During the interviews, the participants underwent both cognitive and affective sense-making (interpretation) of their experiences and we, as researchers, interpreted the participants’ interpretations of their experiences. New understandings were generated in the bridging of the gap between the participant’s frame of meaning and the researcher’s frame of meaning. To successfully achieve this, it was important to have self-knowledge, self-awareness and openness in order to examine our own preconceptions, feelings and motivations in the construction of new knowledge (Frechette et al., 2020; Smith & Smith., 2010).

The analysis at this stage was idiographic and care was taken to ensure that the lived experience of each participant was analysed in detail and valued for its own qualities. The transcripts were read and reread to gain familiarity and detailed explanatory notes were made to support the interpretation of each account. A cross-case analysis was then undertaken to identify convergence and divergence between the participants’ accounts. An approach similar to Braun and Clarke’s (2013) guidelines on thematic analysis was employed at this stage to find commonalities and variability in the data. Keywords and phrases in each transcript were coded and grouped into categories which formed the subthemes within four broad overarching themes which emerged. At this stage, analogical reasoning was employed to make further inferences based on the relational similarity between the cases (Gentner & Smith, 2012). This ensured a detailed and nuanced analysis of the phenomenon under investigation.

Various steps were taken to increase the rigor and reliability of the data analysis. As a team of two (student and supervisor) qualitative researchers, our aim was not to reveal universal truths but to communicate the diverse experiences of the participants and interpret these in their own terms rather than through application of the researchers’ pre-existing theoretical frameworks. The data was collected for a Masters research project and hence the coding was necessarily carried out by the student as primary researcher. Our focus was therefore on intra-coder reliability rather than inter-coder reliability with the emphasis placed on ensuring consistency in how the primary researcher coded data and derived themes at various points throughout the process. We used an approach advocated by Cofie et al. (2022) who recommend at least one of the team, in this case the supervisor, remains external to the process in order to view and examine it with a fresh perspective. Regular discussions between the researchers took place following coding at multiple points in the process with a focus on achieving shared meaning and consensus through reflexive dialogue without losing the authenticity of the participants’ narratives.

## Results

Four key themes were identified and are presented in Table 2 with feeder sub themes.

**Table 2:** Key themes and sub themes

Key Theme	Sub Themes
**1. Children’s experiences and manifestations of trauma are diverse**	Teacher’s and children’s experiences of the pandemic and lockdowns were varied, and they responded in different ways. Children’s behaviours on returning to school following the lockdowns impacted on learning. Diverse and sometimes multiple trauma experiences result in a variety of behavioural responses. Trauma can be experienced at both individual and school community levels.
**2. Trauma management is instinctive and self-motivated with little or no training provided**	Lack of specific trauma training for teachers including within PGCE course. Participants feel ill-informed and untrained but have innate compassion and understanding. Teachers with an interest in trauma are self-motivated to undertake trauma training. Comprehensive training needs to be provided for all. Training needs to be practical as well as theoretical. Having a faith ethos helps provide a framework for managing trauma.
**3. An empathic personality and an environment that is supportive of mental health are important**	Compassionate, empathetic, understanding teachers are paramount - for children and for colleagues. Mental health awareness of both staff and children; a shared trauma. Dealing with children’s trauma can be emotionally exhausting. Having a supportive environment in terms of mental health awareness is important for both staff and children.
**4. Senior management have high expectations but support is inconsistent**	Lack of communication between school staff and leadership in relation to trauma cases. More collaboration is needed between pastoral and teaching departments for best results in the classroom. Lack of support from external agencies due to underfunding and significant time delays which puts teachers under pressure. There needs to be a top-down awareness of the holistic health of the child rather than a data-driven top grades approach.

### Children’s Experiences and Manifestations of Trauma are Diverse

Participants reported encountering a diverse range of childhood trauma during their teaching experiences. Trauma is usually experienced at the individual level and results from experiences such as abuse, neglect and witnessing violence. However, some types of trauma can affect the whole school community as is illustrated below.

“The younger girl was only 18 months when she was removed, she still has memories and feelings but doesn’t know where they come from. The other girl remembers vividly what happened as she was with her parents for longer. She just stands and shakes and is hypervigilant.” (K4, sexual abuse).

“He showed a lot of anger, not physically but verbally aggressive and he had problems with processing and with memory recall.” (C2, trauma following a car accident).

“A few years ago, a Y7 girl slipped under the bus on the way to school. There were children on the bus from all different age groups that saw it happen. Word came that the child was dead on the scene and kids were coming in having seen it. I cried myself when I first got the news, we were dealing with trauma on so many levels that day. Even years later, it still impacts some of the pupils.” (M5, community level trauma).

“She was obviously in deep trauma and a natural response to finding her mother. The pupil was drinking alcohol in school and playing up but after that we thought she might be coming through it. Then, on the night of her mum’s anniversary, she killed herself in the same way her mum did.” (M5, student after finding her mum hanged).

“It all depends on the nature of the trauma but anger, anxiety, aggression, fear, fight or flight mode, social anxieties, problems with eating and hair pulling.” (C2, general behavioural indicators).

“There is a health anxiety among some pupils, they felt that they were going to get Covid-19 or worse, Mummy or Daddy would get it.” (S1).

“I sense an increase in health anxieties; concerns about getting coronavirus themselves or about what happens if the child is living with a single parent – what should happen if my dad dies.” (M6).

“Nearly every day there’s been a fight because they feel lost and disorientated. In addition to that, because they didn’t have to do exams, there’s a sense of loss there too. So, if anything sums the pandemic up its loss and lost.” (M5, student behaviour shortly after schools reopened following post Covid-19 lockdown).

Trauma manifests itself in many different ways as is evident in participants’ accounts. However, children commonly display difficulties controlling their emotions and behaviours, and may also display anti-social behaviours or inflict self-harm. The experience of trauma can affect children’s ability to concentrate and learn in the classroom which can affect their academic attainment in both the short and long term. This presents challenges for teachers who cannot reasonably expect children to behave, react and perform typically in the classroom. The data suggest that far from being infrequent occurrences, manifestations of trauma are common and widespread. Participants who had taught in times of war and civil unrest told how few children in these areas escaped psychological trauma on some level with many children being deeply affected by the death or injury of loved ones. The continuing threat of violence experienced when walking past the aftermath of explosions, hearing gunshots, bombs and emergency vehicles as an almost everyday occurrence is also traumatic. This can be manifested as a suppressed and generalised anxiety for some but for others the continuing experience of multiple traumatic events, sometimes over many years, can result in severe psychological stress and symptoms of post-traumatic stress disorder. Participants also reported the need to be more receptive to changes in children’s behaviour which might indicate possible trauma within the context of the Covid-19 pandemic which was underway at the time the data was collected. Participants told of escalating manifestations of trauma within new and existing cases in the midst of the return to school after the lockdowns.

### Trauma Management is Instinctive and Self-Motivated with Little or No Training Provided

None of the participants had received in-depth training specifically in how to identify the signs and behaviours that might indicate recent or ongoing trauma, or how to manage responses to trauma for individuals or wider school communities. Some participants had been on one-day or half-day general safeguarding courses but felt that this was not enough to provide them with the knowledge and skills needed. Attempts to support traumatised children tended to be based on empathy and instinct, sometimes stemming from participants’ own personal experiences, rather than training. This left some participants feeling uncertain and ill-equipped to support children who were navigating intense and upsetting experiences.

“Often, we pick up on things: demeanour – if a child is off in some way, if they’re normally cheerful, how they dress, the way they present themselves” (T3, describing instincts).

“I don’t think I’ve had any specific training on trauma. It’s just what I’ve picked up from experience.” (C2).

“Your training comes from a lived reality rather than actual trauma training.” (M5)

“If it’s all theory-based then it’s not good. Whereas, if you had more practical training then that would certainly embed it more. Trauma training should be at PGCE level and then there should be regular tasks and activities to keep it up to date.” (K4).

“I’m planning one-hour weekly sessions with a real focus on emotional wellbeing and if anything at all is picked-up we will follow it up. We have talked about the negative impacts of Covid-19 – a Reception child’s dad died from it - and about the experiences of parents who are keyworkers. Now we’re looking at the positives such as new skills they’ve learnt in lockdown and the feelings that have come from it.” (T3, providing emotional wellbeing sessions despite no formal training herself).

“I make sure I talk to the parent every morning – you know the parents who need that extra time because if they’re doing ok then the children have a better chance of doing ok.” (K4, supporting vulnerable families).

“I’ve been phoning some of the families; asking if they have enough food, buying and sending them good books and asking if they’re ok. That connection makes a big difference.” (M5, during the Covid-19 lockdowns).

“I have done some work on the five steps to wellbeing, mindfulness training and mental wellbeing.” (C2, self-motivated learning following the Covid-19 lockdowns).

“We have a way of structuring grief and loss - the faith ethos gives me a framework. I have the gospels to help me so although sometimes they can be sad, there’s always a resurrection story – you don’t have to get stuck in it, but the powers that be don’t see that about faith schools. They fail to recognise there’s a story and a history and a way in which we deal with these life issues that have been part of human existence from the beginning of time.” (M5).

The data presented in above suggest that participants would welcome training that includes practical strategies and theory enabling them to gain knowledge and a range of techniques which can be adapted to each individual child or situation. Participants were aware of several organisations who would come into schools to provide trauma training and felt that these could be utilised to help teachers feel competent and confident in supporting traumatised children. All participants thought trauma training should be embedded within the PGCE year. One participant provided an interesting extra dimension to this by suggesting that trainee teachers could have therapy of their own, as trainee counsellors do, providing the opportunity to reflect upon and grieve their own trauma making them more receptive to trauma indicators in others.

Despite the lack of training, participants described doing their best in the situations they found themselves in. They described how, during the Covid-19 pandemic, they had been self-motivated to provide support for children and families based on instinct, empathy and compassion. They also described how they had been self-motivated in undertaking training courses or facilitated their own learning through accessing resources such as books, webinars and toolkits. Four of the participants either worked or had previously worked in faith schools and described how, in the absence of training, faith could provide a framework to support children with trauma and loss. This was thought to be due to the strong sense of community encountered in religious settings and the role that spirituality can play in aiding trauma recovery by promoting positive coping behaviours, resilience and healing.

### An Empathic Personality and an Environment that is Supportive of Mental Health are Important

Participants spoke of the characteristics they thought teachers needed to enable them to manage traumatised children in the classroom and these included compassion, empathy, understanding and sensitivity. Compassion for what a child has been through along with a deep understanding of the visceral state of a child who has experienced trauma are of paramount importance in helping them feel safe and supported. However, providing this level of support can take its toll on teachers and one participant spoke of feeling emotionally exhausted at times. Another described feelings of inadequacy that surfaced when she started work in a school with children who were facing considerable social and emotional deprivation. These feelings of emotional stress and disempowerment can lead to depersonalisation, cynicism, burnout and the risk that teachers leave the profession. Perhaps unsurprisingly, most participants emphasised their concerns for staff mental health and the need for schools to be more focused on staff wellbeing.

“So, a child loses a parent - that’s trauma and we were very understanding about that – we didn’t have a protocol to follow, just using innate compassion. We just knew that is the way we deal with it…with school staff you will get quite a strong cohort of connected, professional, vocational, compassionate staff and then you’ll get this occasional staff member, ‘And this is the work I said you must do, this is not the time to mess around (or have feelings or stress!!), pull yourself together!’ Showing a rigidity (lack of empathy) in their mindset.” (S1).

“It’s emotionally draining for a teacher if you’ve got many students you have to teach yet maintain a focus on one person (who has experienced trauma). With high achieving schools their primary focus is on the children to get them high achieving and doing well but they also need to look after the teachers. Healthy staff are better teachers.” (C2).

“The teacher or staff member becomes traumatised because they are not fully equipped to understand the child’s experience of life and we end up losing the teacher from the profession - someone who could have been great with the right support.” (S1).

“I became very traumatised by the children’s behaviour and after many years of successful teaching, suddenly I was absolutely disempowered and felt I was failing in what I was doing and I had a breakdown. It took a number of years to actually recover from that sense of failure. I think there is a danger that when children are traumatised and they are acting out, it can feel to teachers almost like it’s a personal failure because they haven’t got enough understanding to know that the child’s behaviour is about their trauma not about the teacher. It’s really important to notice teacher trauma. ‘Hurt people hurt people’ – even if unintentionally. If we offer teachers compassion, care and support it filters down.” (S1).

The data within this theme strongly suggests that schools need to support teachers mental health and wellbeing in order to help them cope with the manifestations of trauma that they encounter within the classroom. With effective support, the distress of burnout might be avoided, teachers will be enabled to perform their jobs to their full potential, and schools might retain valuable members of staff.

### Senior Management have High Expectations but Support is Inconsistent

This theme arose from participants’ reflections on their concerns and anxieties in relation to the support that they receive from senior management. They reported that senior leadership teams do not always communicate information about known cases of trauma which can affect the way teachers manage children in the classroom. Participants understood that there were confidentiality and data protection issues at stake but despite this, it was felt that communication could be improved.

It also emerged that external agencies struggled to cope with the high referral rate of trauma cases in the wake of the pandemic leaving participants with concerns about children’s access to support services. Agency working practices, underfunding and time-delays were all perceived as barriers in providing support to parents and children in need. A further barrier to receiving support was where the school had identified a need, but the parents were reluctant to accept help. All of this led to some mixed feelings among participants about the helpfulness and usefulness of external support services.

“We’ve been having issues with senior management where they’re not telling us what’s happening with the children in our class. We don’t need to know the details, but we still need to know if there’s any issues as this would change the way we manage them.” (T3).

“There’s a close symbiotic relationship between the pastoral and academic side but in some schools, you would think they are two separate things.” (M6).

“We need a middle-management team; this is the pastoral struggle and this is the academic struggle. Let’s build a new narrative and work together to talk and inform each other – any tool that deals with where a person is, will ultimately help them in their education but to see them as separate is dangerous.” (M5).

“There seems to be no actual help for the mental health of the child. If they (social services) think the child’s parents or foster parents are safe, then that’s enough. What the child has gone through seems to be ignored and swept under the carpet.” (K4).

“Education and social services are underfunded. We can’t always signpost our children properly as there’s no-one to pick it up.” (T3).

“Social workers tend to drop in and drop out then go, so foster carers are really on their own (with traumatised children). Also, last October (7 months ago) we referred a family on for help and they still haven’t heard anything – nothing is happening from social services end due to the pandemic.” (K4).

“With one agency, they’ll come in and we can give them the names of the children that need help – but only if parents agree to it. Sometimes the parents say they don’t need help or support and that’s a bit worrying.” (T3).

“It seems barmy that we have to get the parents’ permission when we know what they need, surely it should be our professional opinion, but we need the parents’ consent. The system isn’t helping these children.” (K4).

“Even when the class went swimming, I had to be in the changing room with him as he couldn’t be trusted. He couldn’t even go to the toilets on his own. Then one day he pushed me away and threatened to stab me with a knife – did the same with his foster family, he went from carer to carer after that. He only had counselling every few months and I remember thinking it seems a bit haphazard then. This was someone in pain who would lash out at anybody and wasn’t getting the help he needed.” (K4).

“What worries me in education at the moment is that there is a rigid, outcomes and data-driven environment and it is top down. There’s no question about it. The government is putting pressure on the Education Department, which in turn is putting pressure on academies, schools or county councils to get the right results. The seeking of excellence is not about top grades but…what is good for the whole of the child.” (S1).

The data highlighted a separation between the pastoral and academic departments within schools and participants had concerns about this, pointing out that traumatised children are often in survival mode or self-protective mode meaning they are not fully present emotionally in the classroom and find it difficult to apply themselves to learning. This problem was exacerbated by the top-down, data-driven approach of school leadership teams and government who were perceived to prioritise academic results whilst failing to consider the holistic needs of the child.

## Discussion

The findings indicate that childhood trauma is a common occurrence and manifests itself in various ways within the classroom. Participants’ accounts illustrate how a single traumatic episode can result in a complex post-traumatic stress response which can include difficulties with concentration; difficulties controlling emotions and behaviour; and a range of anti-social and self-harming behaviours. This can present significant challenges, both for the children experiencing trauma and their teachers, affecting children’s development, educational progress and attainment, findings which are consistent with a number of other studies (Bethell et al., 2014; Duke et al., 2010; Perfect et al., 2016; Stempel et al., 2017). Although the sample is small, the data suggest that children’s experiences of trauma are not infrequent occurrences but are widespread and wide-ranging. Teachers might come across single or multiple cases in any given day and are required to navigate through them and support children’s recovery within the context of their general classroom management, often without specific or in-depth training.

Participants recounted how some pupils had experienced multiple losses, particularly those living through periods of civil war and unrest. In some cases, children were uprooted from their homes and faced pressures to assimilate a new culture causing further loss and a sense of displacement, findings mirrored by Iraklis (2021). These experiences were mainly those of participants who had taught in Namibia during the border wars and Northern Ireland during the Troubles and as part of the analysis it is important to acknowledge factors which might influence the results. However, these experiences are particularly pertinent and have the potential to inform practice in the current context of the war between Russia and Ukraine. At the time of writing 11,400 Ukrainian children had applied for places in British schools having sought refuge from the war in Ukraine and this number is continuing to rise (DfE, 2022b). According to DfE (2019), a total of around 613,000 foreign-born children moved to the UK and started in British schools. Children aged 9 or under were found to have similar attainment levels at the end of KS4 compared to their UK born peers but those arriving after age 12 had significantly lower attainment levels than their peers suggesting that younger children have fewer difficulties integrating into a new culture, language and home. These statistics along with the qualitative findings from this study support Paat’s (2013) recommendation that a focused and individualised approach to trauma management would be beneficial in supporting resettlement of refugee and other immigrant children.

Trauma management within classrooms tends to be instinctive, reactive and based on human instincts such as compassion, empathy and understanding. Participants conveyed a sense of firefighting in their accounts of dealing with children’s trauma both in the past and more currently amid escalating cases within the context of the Covid-19 pandemic. In the absence of specific training, support and protocols teachers undertook self-facilitated and self-motivated learning and research to equip themselves with relevant skills and knowledge to support children in the classroom. As a result, participants had awareness of a range of symptoms, signs and behaviours that traumatised children might exhibit. However, they felt that a full and comprehensive training programme was needed to help them to develop more fully the knowledge and skills needed to support the children in their care. They made suggestions for what should be included in the training including a mix of theory and a range of strategies and techniques they could draw upon and adapt to tailor their approach to each individual child. One teacher who is also a counsellor suggested that trainee teachers could have therapy of their own, as trainee counsellors do, providing the opportunity to reflect upon their own experiences of trauma which would help to support their own mental health, reduce their vulnerability in dealing with trauma in the classroom and develop a more informed and personal understanding of children’s adverse childhood experiences. These findings build on the existing literature which points to a considerable lack of trauma training available for teachers leading to feelings of uncertainty and being ill-equipped (Alisic, 2012; Asmussen et al., 2019; Berens et al., 2017; Reinke et al., 2011; Teicher et al., 2016; Wing et al., 2015). Without comprehensive training, there is a possibility teachers will miss trauma and fail to identify and provide appropriate support which in some cases could lead to severe and tragic outcomes.

The participants in this study emphasised that teachers would find a whole-school strategy and approach to dealing with the effects of trauma beneficial and supportive. This is consistent with a study by Signorelli et al. (2017) who found that successful trauma management should involve relationship building with the entire school community, along with a commitment to shared goals. Participants felt that it was important for schools to promote a sense of normality particularly during times where the whole school is affected by events such as during the Covid-19 pandemic. They also suggested that schools could adopt neurological repair methods such as mindfulness training. This is supported in a study by Bryck and Fisher (2012) which found that mindfulness training is a good way to mediate the impact of early life stress and anxiety. Two thirds of the participants had experience of working in faith schools and they spoke of feeling more confident in managing the various types of traumas they encountered within a faith perspective. This was thought to help children feel more secure due to the strong sense of community often present within faith schools and the shared cultural understanding of staff, children and families. This idea is supported by Bryant-Davis and Wong (2013) and Van Dyke et al. (2009) who found that faith-based coping strategies and access to support from faith communities aids trauma recovery and can lead to an increased sense of wellbeing, resilience and healing. However, whether the school is faith-based or not, positive role-models and supportive, understanding adults can enable positive change in children’s ability to cope and recover from trauma, and to improvements in self-esteem and academic achievement (Herrmann et al., 2016; Silcox, 2021; Yancey et al., 2002).

Participants’ narratives of shared trauma, sometimes impacting the whole school, illustrate how teachers can find themselves in particularly challenging and demanding positions requiring them to be dually aware of their own mental health needs and those of the children. Dealing with trauma on a daily basis can bring significant risks to teachers mental health and wellbeing including depression, burnout, re-traumatisation, secondary traumatic stress, depersonalisation and a sense of professional or personal inadequacy (Schaufeli et al., 2009). Experiencing these things makes it more likely that teachers will leave the profession contributing to the current teacher retention crisis. The findings of this study suggest that, in order to avoid the distress of burnout and loss of valuable members of staff, schools should balance high expectations with effective support and implement approaches and interventions focused on protecting and supporting teachers’ mental health. There is a growing body of research which supports this, pointing to the positive effects of employing emotional intelligence competencies in managing stress, identifying the symptoms of burnout and supporting regulation of emotional experiences that may contribute to burnout (Austin et al., 2008; Karimi et al., 2014; Saklofske et al., 2012; Ullrich et al., 2012; Zysberg, 2017).

A number of participants raised the issue that, for data protection reasons, senior leadership teams were not always forthcoming in communicating information about known cases of trauma to classroom teachers. This can affect teachers’ understanding of and responses to the behaviours they encounter in the classroom and delay referral of vulnerable children and families to external agencies such as mental health services. When children were referred, participants reported that there was variable success in accessing support possibly due to a surge of pandemic-related referrals leaving services underfunded and understaffed, and children at risk. This is supported by Henderson et al. (2020) who found that external agencies are struggling to cope with the demand of pandemic mental health and trauma referrals. Addressing this issue is imperative to improving the protection and recovery of struggling families as well as enabling teachers to feel more confident and supported in their roles.

Chafouleas et al. (2016) and SAMHSA (2014) report that, despite a growing awareness of the toll of trauma exposure on children’s outcomes and the benefits of early intervention, schools do not do enough to address issues related to trauma such as child maltreatment or family dysfunction. The participants in this study suggested that, in addition to improving communication between senior leaders and classroom teachers, one thing schools could do to improve their response is to have a joined-up approach to their pastoral and academic support. This has also been highlighted by Best (2014) and Cahill et al. (2010) who found that integrated pastoral and academic support services can enhance the overall educational experience, leading to improved support and academic achievement.

It is important to point out that the experiences of the teachers in this study may or may not reflect the experiences of others. Nonetheless, their experiences resonate with the findings of many other studies and provide additional insights into how manifestations of childhood trauma are managed within schools during the first wave of the coronavirus pandemic. Readers will be able to judge for themselves the potential transferability of findings to their own contexts and settings.

## Conclusion

No firm conclusions can be drawn from the findings of this study given the small sample, but the indications are that childhood trauma occurs frequently and manifests itself in various ways within the classroom. Supporting children in the classroom presents challenges for teachers who are required to manage children’s emotional and behavioural responses to trauma which can affect their progress, attainment and long-term outcomes. Dealing with classroom-based childhood trauma with little or no training also brings risks to teachers’ mental health and can contribute to feelings of depression, a sense of professional inadequacy and burnout, increasing the likelihood of teachers leaving the profession. Training is needed at the beginning and throughout teachers’ careers to help them develop the skills and knowledge needed to support traumatised children in the classroom effectively. Clear communication from leadership teams to identify trauma-affected pupils to their teachers is also needed as this informs how teachers manage behaviour and enables them to adopt an empathic and compassionate approach. Adopting a whole-school trauma-informed strategy which includes neurological repair methods such as mindfulness training and an integrated approach to academic and pastoral provision can improve support for traumatised children. Therefore, schools might consider evaluating and updating their strategies, approaches and policies for managing trauma to provide effective support for children and staff.

### Research Limitations

The study limitations are the limitations of the IPA method: small, homogenous sample, difficulty in making generalisations and subject specificity.

A future study could extend the homogenous sample to achieve a greater degree of generalisability and correlations, exploring a single theme: specifically post-pandemic trauma support for schoolchildren and their respective teachers and support staff.
